# Genome-Wide Identification and Characterization of Heat Shock Proteins in the Stored-Product Pest *Rhyzopertha dominica* (Fabricius): Phylogenetic, Structural, and Stress-Induced Expression Analyses

**DOI:** 10.3390/insects16020127

**Published:** 2025-01-28

**Authors:** Yueliang Bai, Yanzhu Xie, Junji Yao, Fangfang Zeng, Dianxuan Wang

**Affiliations:** 1 Grain, Oil and Food Engineering Technology Research Center of the State Grain and Reserves Administration/Key Laboratory of Henan Province, Henan University of Technology, Zhengzhou 450001, China; 2 National Grain Industry (Storage Insect Pest Control) Technology Innovation Center, School of Food and Strategic Reserves, Henan University of Technology, Zhengzhou 450001, China

**Keywords:** *Rhyzopertha dominica*, heat shock proteins, stored-product pests, stress response, expression pattern

## Abstract

Heat shock proteins (HSPs) are crucial molecular chaperones that help organisms maintain protein stability under stress conditions. As a major stored-product pest, *Rhyzopertha dominica* (Fabricius) faces distinct stresses compared to field insects, primarily due to the specific pest control methods applied during grain storage. In this study, a total of 53 HSP genes from five gene families (HSP90, HSP70, HSP60, sHSP, and DnaJ) were identified and characterized using bioinformatics methods. Among them, DnaJ was the largest and the most diverse HSP family in *R. dominica*. Transcriptome sequencing and RT-qPCR were then used to evaluate HSP gene expression patterns under four storage-related stresses, following a series of bioassays. Extreme high temperature was the strongest inducer of HSP expression, with 12 genes showing over a 10-fold increase. Controlled nitrogen atmosphere also led to considerable upregulation of HSP genes, especially in the HSP70 family. In contrast, phosphine fumigation and K-Obiol grain protectant caused very limited induction of HSP genes, which might have been due to the less severe protein damage caused by chemical stresses compared to physical stresses. Our study provides a theoretical basis for further research on HSP functions in *R. dominica*.

## Simple Summary

The lesser grain borer, *Rhyzopertha dominica* (Fabricius), is a major pest of stored grains worldwide, causing significant economic losses every year. It not only damages various stored cereals and legumes, but also stored materials like wood, paper, and leather, exhibiting a wide range of severe destructiveness and strong environmental adaptability. Heat shock proteins (HSPs) are known to play a vital role in stress adaptation, which can help *R. dominica* cope with various environmental challenges. In this study, 53 HSP genes from the HSP90, HSP70, HSP60, sHSP, and DnaJ families were identified in the *R. dominica* genome, among which the DnaJ family was the largest and most diverse HSP family. Physical stresses, such as extreme high temperature and controlled nitrogen atmosphere, induced strong upregulation of HSP expression compared to chemical stresses, such as phosphine fumigation and K-Obiol grain protectant treatments. This research provides a basis for further studying the function of HSP genes in R. *dominica*, and offers valuable molecular resources for developing specific control strategies for stored-product pests.

## 1. Introduction

Heat shock proteins (HSPs) are a conserved family of proteins that were initially discovered in *Drosophila melanogaster* and named for their ability to be induced at high temperatures [1]. Acting as molecular chaperones, HSPs protect proteins from various stress conditions by preventing misfolding and aggregation, while also promoting refolding or degradation of damaged proteins [2,3]. Current studies have shown that HSPs can be rapidly induced by a wide range of stresses, such as extreme temperatures [4,5], insecticides [6], heavy metals [7], UV radiation [8], etc. [9,10], and they are considered to play important roles in helping organisms, including insects, resist negative environmental conditions [11,12].

Based on molecular weight, sequence homology, and function, insect HSPs are classified into several major families, including HSP90, HSP70, HSP60, and the small HSPs (sHSPs). The HSP70 family has been particularly well studied, and members of this family play critical roles in protein folding, translocation, and prevention of stress-induced protein aggregation [13]. They are widely present in prokaryotes and eukaryotes, and are thought to be associated with the response to various stresses [14,15]. HSP90 is one of the most abundant cytosolic proteins in eukaryotic cells and is crucial in maintaining proteostasis [16]. It also contributes to processes like suppressing mobile genetic elements, regulating epigenetic modifications, DNA repair, and immune response regulation [17,18,19]. The HSP60 family is primarily responsible for the proper folding of newly synthesized proteins within mitochondria, and thus are involved in maintaining proteome integrity and homeostasis, and also function as housekeeping genes [20,21]. The small HSPs, members of the HSP20 family, are a group of low molecular weight chaperones that primarily function to prevent protein aggregation under extreme environmental conditions [22,23,24]. Their versatility and rapid induction make them particularly important in the early stages of stress responses [25,26].

In addition to the well-known heat shock protein families, the DnaJ family (also known as HSP40) plays a key role in cellular stress responses and basic physiological processes [27]. As co-chaperones of HSP70, DnaJ proteins assist in crucial processes like protein folding, degradation, and translocation by stimulating the ATPase activity of HSP70 through their conserved J-domain [28,29]. Beyond this conserved domain, DnaJ proteins exhibit considerable structural diversity and can be further classified into three subfamilies, DnaJA, DnaJB, and DnaJC, based on differences in their domain organization [30]. This structural diversity gives them a broader range of functions to participate in a wide range of stress-related processes. However, the systematic identification and functional studies of DnaJ proteins in insects are still limited compared to other HSPs.

The lesser grain borer, *Rhyzopertha dominica* (Fabricius) (Coleoptera: Bostrichidae), is a major stored-product pest that is widely distributed across the world, with a significant destructive capacity for stored grains and dried products such as rice, wheat, sorghum, soybeans, wood, and other commodities [31,32,33]. Both larvae and adults of *R. dominica* have well-developed mandibles that allow them to damage both intact and broken grains, resulting in severe weight loss [34,35]. A single grain kernel can be bored and damaged by more than one *R. dominica* individual, eventually leaving only an empty shell. In addition to directly reducing grain weight, *R. dominica* infestation can also increase the levels of dust and frass within the grain mass, which can promote the proliferation of secondary pests and microorganisms, reduce grain pile porosity, and subsequently lead to increases in moisture and temperature, seriously threatening the stability and safety of grain storage [36,37,38,39].

Over the years, fumigants, protectants, and other chemical agents have been used to control *R. dominica*. However, continuous and improper application of these chemicals has resulted in significant resistance in this pest [40,41,42], which might eventually lead to the complete failure of chemical control. Consequently, in recent years, environmentally friendly physical control methods such as temperature treatment and controlled atmosphere have gradually begun to replace chemical treatments [43,44]. However, studies have shown that, with extended exposure, the adaptation of pests to these physical control methods may also increase [45]. Therefore, understanding the resistance mechanisms of pests to common grain storage control methods can provide valuable insights for more effective management.

Given the crucial role of heat shock proteins in stress responses and adaptation, they have been extensively studied in agricultural and public health pests, but systematic research on these genes in stored-product insects remains limited [24,46,47,48]. Since the environmental pressures experienced by stored-product pests often differ from those affecting field pests, it is essential to systematically identify the HSPs in stored-product pests and explore their response patterns under these specific stress conditions. As a major pest in grain storage, *R. dominica* is highly adaptable to diverse environmental stresses, making it an ideal model for studying the role of HSPs in stress resistance. Previous molecular studies on *R. dominica* have primarily focused on insecticide resistance genes such as *rph1* and *rph2* [49,50]. However, with the increasing adoption of environmentally friendly physical control methods in grain storage, such as controlled atmosphere, temperature regulation, and ionizing radiation, it is important to understand the processes of stored-product pests in response to physical stresses. As key molecular chaperones, HSPs play important roles in helping insects cope with the protein damage and cellular dysfunction caused by physical stresses such as high temperatures and low oxygen. In addition to their role in physical stress resistance, HSPs can also respond to certain chemical stresses. However, research on the HSP genes of *R. dominica* remains limited.

In this study, we performed a comprehensive identification of HSP genes from the HSP90, HSP70, HSP60, sHSP, and DnaJ families in the *R. dominica* genome, followed by an in-depth analysis of their genomic locations, gene structures, conserved motifs, and protein characteristics. We further assessed its tolerance to four stored-product pest control strategies: controlled nitrogen atmosphere, extreme high temperature, phosphine fumigation, and K-Obiol grain protectant treatments. Subsequently, based on the LT_50_ and LC_50_ values of each treatment, we further carried out transcriptome sequencing and RT-qPCR to measure the expression patterns of the HSP genes. Beyond offering insights into the stress response mechanisms in *R. dominica*, our study also provides valuable molecular resources for investigating resistance mechanisms in stored-product pests, contributing to the development of more effective pest control strategies.

## 2. Materials and Methods

### 2.1. Insect Rearing

The *R. dominica* population used in the experiment was originally collected from a grain storage facility in Nanyang, Henan Province, China. Approximately ten pairs of adults were isolated from the collected infested wheat under a microscope. The population was then continuously reared for more than 20 generations in an incubator with a temperature of 30 ± 1 °C, a relative humidity of 70 ± 5%, and under a 24 h dark photoperiod. No chemical agents or physical stresses were applied for resistance selection during this period. The insects were reared on a mixture of intact and broken wheat grains as feed. The openings of the rearing bottles were evenly coated with polytetrafluoroethylene (PTFE) and covered with filter paper and mesh to prevent insect escape.

### 2.2. Genome-Wide Identification of the HSP Genes

Although the genome of *R. dominica* has been sequenced and assembled (GenBank BioProject ID: PRJNA449115) in 2022 [51], a whole genome annotation of this pest has not been published. Therefore, we first performed an automated annotation of the whole genome through the modified OMIGA (Optimized Maker-Based Insect Genome Annotation) pipeline [52], followed by manual correction and supplementation of each HSP family gene. The specific steps were as follows: Firstly, the protein sequences of HSP genes of *D. melanogaster*, *Bemisia tabaci*, and *Tribolium castaneum* were downloaded and used as query sequences for BLASTP (version 2.10.0+) against the genome protein sequences of *R. dominica* with a cutoff of 1 × 10^−5^ to obtain the preliminary candidate HSP genes based on sequence similarity, and reciprocal best hits were considered for ortholog assignment. HMMER (version 3.3.2) [53] was further employed to identify structural domains in the putative sequences obtained in the previous step by searching against the Pfam database. Sequences containing specific Pfam motifs (HSP90: PF00183, HSP70: PF00012, HSP60: PF00118, HSP20: PF00011, DnaJ: PF00226) were considered candidate family members. For candidate genes with incomplete sequences from the automated pipeline, 5–10 kb upstream and downstream genomic DNA sequences were extracted based on their annotation and further annotated using the Fgenesh and Fgenesh+ online tools [54] (http://www.softberry.com/berry.phtml?topic=case_study_animal&no_menu=on/, accessed on 12 August 2024) to obtain full sequences. Finally, the annotation accuracy and sequence completeness of all candidate HSP members were verified based on alignment results (similarity, coverage, and length) with homologous sequences from the Nr database (NCBI Non-redundant Protein Sequence Database), and the presence of conserved domains was confirmed by searching the NCBI Conserved Domain Database (http://www.ncbi.nlm.nih.gov/cdd/, accessed on 12 August 2024).

### 2.3. Bioinformatics Analysis of HSP Genes

The molecular weight and theoretical isoelectric point of the identified HSP genes of *R. dominica* were analyzed using ExPASy (https://web.expasy.org/protparam/, accessed on 20 August 2024). Subcellular localization was predicted by CELLO (http://cello.life.nctu.edu.tw/, accessed on 20 August 2024). Gene structure analysis was conducted with GSDS (http://gsds.gao-lab.org/, accessed on 22 August 2024), and conserved motif discovery was performed using the MEME Suite (https://meme-suite.org/meme/tools/meme/, accessed on 24 August 2024).

For phylogenetic analysis, all the putative HSP protein sequences were first aligned using MAFFT (version 7.475) [55] and trimmed by trimAl [56] (version 1.4.rev15) with default parameters. Then, a maximum-likelihood phylogenetic tree was constructed using RAxML (version 8.2.12) [57] with 1000 bootstrap replicates based on the best-fit model predicted by ProtTest (version 3.4) [58]. Finally, the phylogenetic tree was visualized with the iTOL online tool (https://itol.embl.de/, accessed on 28 August 2024) [59]. For phylogenetic analysis of the HSP genes among different species, we obtained reliable HSP protein sequences from various insect species, including *D. melanogaster*, *Aedes aegypti*, *Bombyx mori*, *B. tabaci*, *Nilaparvata lugens*, *T. castaneum*, and *Anoplophora glabripennis*. These HSP sequences were downloaded from FlyBase (http://flybase.org/, accessed on 24 August 2024), SilkDB (https://silkdb.bioinfotoolkits.net/, accessed on 24 August 2024), NCBI (www.ncbi.nlm.nih.gov/, accessed on 24 August 2024), and references [15,47,60,61]. All the sequences used in this study can be found in Data S1 and Data S2.

### 2.4. Controlled Nitrogen Atmosphere Treatment

First, 30 adult *R. dominica* one week after emergence were randomly selected and placed in a glass tube with mesh-covered ends. Three tubes were then transferred together into a 1100 mL gas washing bottle. To prevent the insects from starving during the experiment, 0.3 g of cracked wheat was added to each glass tube beforehand. Both ventilation ports of the gas washing bottle were tightly connected to latex tubes of the same diameter, with Vaseline applied at the connection points to ensure no air leakage. One end of the latex tube was connected to a gas cylinder to introduce nitrogen, while the other end was used for exhaust or connected to a gas detector. After adjusting the nitrogen concentration inside the gas washing bottle to 99 ± 0.5%, both ends of the latex tubes were clamped. No nitrogen was introduced into the gas washing bottles of the control treatment. Every 24 h, two gas-washing bottles (one from the control group and one from the treatment group) were randomly selected. All test insects were removed from the bottles, and their mortality was observed and recorded. The corrected mortality rate was calculated using the following formula: Corrected Mortality Rate = [(Mortality Rate of Treatment Group – Mortality Rate of Control Group)/(1 – Mortality Rate of Control Group)] × 100%. The median lethal time (LT_50_, Time) value was obtained by fitting logistic regression using Origin software (version 2022).

### 2.5. Extreme High Temperature Treatment

Elevated temperatures have been considered an effective method for managing stored-product pests, especially short extreme high temperature treatment. To test the tolerance of *R. dominica* to the high temperature in a short time period, mortality was tested at different temperatures for 3 h. Thirty insects (one week after emergence) were first transferred into a mesh-covered glass tube. The upper half of the inner wall of the glass tube was coated with PTFE to prevent the insects from climbing. The bottom two-thirds of the tube were then completely immersed in a water bath, ensuring that the temperature in the area where the insects were located quickly reached the set temperature. Insects were exposed to temperatures of 30 (control), 34, 38, 42, 44, 45, 46, 47, 48, and 49 °C for 3 h, then their mortality was calculated immediately and after a 24 h recovery. Three biological replicates were performed for each treatment. The corrected mortality rate of each temperature (with 24 h recovery) was calculated and the median lethal temperature (LT_50_, Temp) was determined by fitting logistic regression using Origin (version 2022) software (OriginLab Corporation, Northampton, MA, USA).

### 2.6. Phosphine Fumigation Treatment

A phosphine fumigation experiment was conducted according to the FAO method No.16 (Food and Agriculture Organization 1975) with some modifications. Thirty insects (one week after emergence) were placed in a mesh-covered open-ended glass tube, and then three tubes were transferred together into a 1100 mL triangle gas washing bottle (the same as the equipment used in the controlled nitrogen atmosphere treatment) before the introduction of phosphine. Different volumes of phosphine were then introduced to different prepared bottles to create a series of treatment concentrations. At the same time, a phosphine gas detector was connected to the bottles to measure the phosphine concentration and adjust it to the set concentration. Based on the pre-experiment, the phosphine treatment concentrations were set at 0 (control), 0.15, 0.45, 0.75, 1.05, and 1.35 g/m^3^. After 20 h of treatment followed by 14 days of incubation under normal conditions, mortality was observed and recorded. Corrected mortality rates were calculated and the median lethal concentration (LC_50_) value was determined through Probit regression analysis.

### 2.7. K-Obiol Grain Protectant Treatment

K-Obiol (Bayer CropScience, Hawthorn East, Australia), a widely used grain protectant with deltamethrin as the active ingredient (25 g/L), was selected for use in the grain protectant experiment. Firstly, K-Obiol was diluted to different concentrations based on the active ingredient and 1 mL of each dilution was applied evenly onto filter paper. After air drying, the treated filter paper was transferred to a petri dish with the same diameter (8.5 cm), and the inner wall of the petri dish was coated with PTFE to prevent the test insects from escaping. After that, 30 insects (one week after emergence) were placed in the peri dish, and mortality was observed after 24 h. Based on the pre-experiment, the concentrations of the active ingredient used in this experiment were set at 0 (control), 0.0625, 0.125, 0.25, 0.5, 1, and 2 µg/cm^2^. Three replicates were used for each treatment. Corrected mortality rates were calculated and the LC_50_ value was determined through Probit regression analysis.

### 2.8. Transcriptome Sequencing and Expression Pattern Analysis of HSP Genes

Firstly, RNA was extracted using Trizol reagent (Thermo Fisher Scientific, Waltham, MA, USA) from surviving insects (one week after emergence) following treatment with fumigants and grain protectants at LC_50_, as well as the controlled nitrogen atmosphere LT_50_ (Time). Since the insects entered a state of heat coma after exposure to the LT_50_ (Temp), to avoid the effects of this condition and unidentifiable dead insects on gene expression, we selected active insects (not in coma) after 3 h of treatment at 46 °C for RNA extraction. Three replicates for each treatment (or control) and 30 individuals for each replicate were considered. After that, RNA integrity was assessed using the RNA Nano 6000 Assay Kit of the Bioanalyzer 2100 system (Agilent Technologies, Santa Clara, CA, USA). In total, 24 RNA samples from 4 treatment groups were prepared for library construction.

A total amount of 1 µg RNA per sample was used to prepare RNA libraries using a Fast RNA-seq Lib Prep Kit V2 (ABclonal, Wuhan, China) following the manufacturer’s instructions. mRNA was enriched from total RNA using a poly (A) mRNA Capture Module (ABclonal, Wuhan, China). After fragmentation, the first strand cDNA was synthesized using random hexamer primers, followed by second strand cDNA synthesis. After end repairing, A-tailing, adapter ligation, size selection, amplification, and purification, the library was further checked with Qubit and real-time PCR for quantification and a bioanalyzer for size distribution detection. Then, samples were clustered using a TruSeq PE Cluster Kit v3-cBot-HS (Illumina, San Diego, CA, USA) and sequenced on the Illumina Novaseq platform at Novogene (Beijing, China) with the PE150 strategy. After removing adapters, low-quality reads, and high-content unknown base sequences using fastp (version 0.23.2) [62], clean reads were obtained and the Q20, Q30, and GC contents were calculated. Next, clean reads were mapped to the reference genome using HISAT2 (version 2.0.5) [63], and the read numbers mapped to each gene were counted and transferred to FPKM using the featureCounts tool [64] from the Subread software (version 1.5.0-p3) [65]. The expression level of each gene under non-stressed conditions was calculated by averaging the FPKM values from 12 control samples (CN1, CN2, CN3, CH1, CH2, CH3, CP1, CP2, CP3, CK1, CK2, and CK3). Fold changes between control and treatment conditions (three biological replicates per condition) for each comparison group were calculated using DESeq2 (version 1.20.0) [66], and the log2-transformed fold changes were used to generate a heatmap. DESeq2 also provided statistical routines for determining differential expression in gene expression data using a model based on the negative binomial distribution. The resulting *p*-values were adjusted using the Benjamini–Hochberg (BH) approach to control the false discovery rate. Genes with adjusted *p*-value ≤ 0.05 and |log_2_(fold change)| ≥ 1 were assigned as differentially expressed. Gene ontology (GO) enrichment analysis of the differentially expressed genes was implemented using the clusterProfiler R package (version 3.8.1) [67]. GO terms with a corrected *p*-value less than 0.05 were considered significantly enriched by differential expressed genes. All the results were visualized using the OmicShare online tool (https://www.omicshare.com/tools/, accessed on 28 August 2024) [68] and Adobe Illustrator software (version 2024).

### 2.9. RT-qPCR Verification

RT-qPCR was used to validate the reliability of the transcriptome analysis results. Six HSP genes were selected for RT-qPCR verification, including *RdHSP90-1*, *RdHSP70-1*, *RdHSP60-1*, *RdsHSP21.0*, *RdDnaJ-3*, and *RdDnaJ-7*, with *RdEF1α* and *RdRPS6* as reference genes. The same RNA samples as used for transcriptome sequencing were used as templates. The RNA from each sample was first reverse-transcribed into cDNA using Script III All in one RT Mix With dsDNase (GeneBetter, Beijing, China) according to the manufacturer’s instructions. Then, the reversed cDNA was used as a template on a Bio-Rad CFX96 Touch Real-Time PCR Detection System (Bio-Rad Laboratories, Hercules, CA, USA) with 2 x Universal SYBR Green qPCR Mix (GeneBetter, Beijing, China) for qPCR detection. The primers (Table S1) used in the experiment were designed with Primer3 (https://bioinfo.ut.ee/primer3-0.4.0/ accessed on 20 November 2024) [69]. Finally, the relative expression levels of genes were calculated using the 2^−∆∆CT^ method [70] with two reference genes. The Pearson correlation coefficients between the RNA-Seq and RT-qPCR results for each gene were calculated using SPSS (version 29.0.1.0).

## 3. Results

### 3.1. Genome-Wide Identification of HSP Genes in R. dominica

A total of 53 HSP genes were identified and manually corrected from the *R. dominica* genome. By searching against the Pfam database, 53 genes were classified into five gene families: three belonging to the HSP90 family, ten to the HSP70 family, one to the HSP60 family, nine to the sHSP family, and 30 to the DnaJ family. The identified HSP genes of *R. dominica* were abbreviated using the initials of its scientific name, followed by the gene family name and then the gene number. *RdHSP70s* and *RdHSC70s* represent the inducible and constitutive members of the heat shock protein 70 family, respectively. For genes in the sHSP family, the gene names end with their molecular weight. Since *RdHSP90-3* possesses the typical structural domain and high sequence similarity, we retained it even though the sequence was incomplete.

The molecular weight, theoretical isoelectric point (pI), subcellular localization, and additional characteristics of the HSP90, HSP70, HSP60, and sHSP genes are presented in Table 1. Due to the large size of the DnaJ gene family, their corresponding information is provided separately in Table 2. As shown in Table 1, the average molecular weight is lowest for the sHSP gene family (19.6 kDa–21.9 kDa) and highest for the HSP90 gene family (82.3 kDa–86.1 kDa). Based on the subcellular localization prediction results, most genes from the HSP90 and HSP70 families are located in the cytoplasm, with a few also present in the nucleus, mitochondria, and endoplasmic reticulum. The HSP60 gene is predicted to be located in the mitochondria, while sHSPs might be located in the nucleus, mitochondria, cytoplasm, or extracellular area.

Based on similarity alignment with known DnaJ genes from other species, 30 DnaJ genes were identified in *R. dominica*, of which three belong to the DnaJA subfamily, six to the DnaJB subfamily, and 21 to the DnaJC subfamily (Table 2). Compared to the other HSP genes, genes from the DnaJ family show greater variation in basic physiological and biochemical characteristics. Even though they are also known as HSP40s, the molecular weights of these genes range from 15.6 kDa to 254.7 kDa. Based on the subcellular localization results, most DnaJ family members are predicted to be located in the nucleus, followed by the cytoplasm, with a few members also found in the mitochondria, plasma membrane, and other regions.

### 3.2. Phylogenetic Analysis

**Figure 1:**
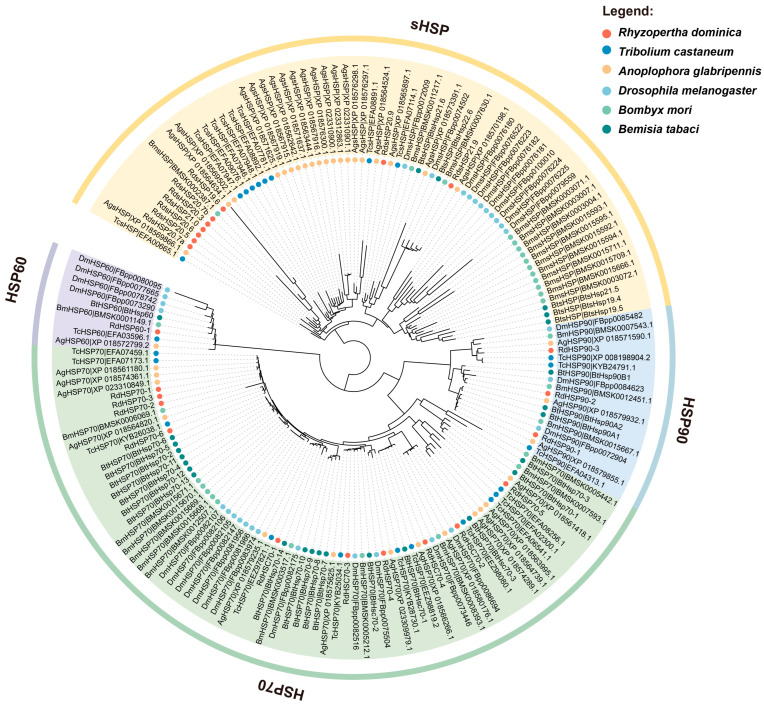
Phylogenetic analysis of HSP90, HSP70, HSP60, and sHSP family members from *Rhyzopertha dominica* and other species. The maximum-likelihood phylogenetic tree was constructed using RAxML with 1000 bootstraps. The colored shading and dots mark the different HSP families and species, respectively.

To further understand the evolutionary relationships between the members of the heat shock protein superfamily in *R. dominica* and other species, we constructed a maximum-likelihood phylogenetic tree based on the protein sequences of HSP genes. As shown in Figure 1 and Figure S1, the HSP genes of the six species were perfectly grouped into HSP90, HSP70, HSP60, and sHSP branches, reflecting the overall conservation of these protein sequences across species. The clustering results of all *R. dominica* HSP sequences were consistent with the gene family predictions from our Pfam search based on hidden-Markov models, further validating the accuracy of the identification. However, the branch lengths of sHSP genes were relatively longer than those of other families and showed a significant species-specific expansion pattern. Among the six species, the HSP proteins of *R. dominica* clustered more closely with those of *T. castaneum* and *A. glabripennis*, consistent with their taxonomically closer evolutionary relationships.

**Figure 2:**
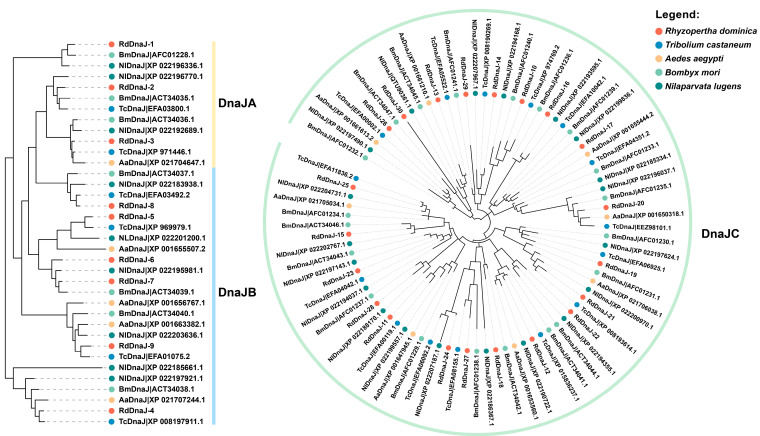
Phylogenetic analysis of DnaJ family members from *Rhyzopertha dominica* and other species. The maximum-likelihood phylogenetic tree was constructed using RAxML with 1000 bootstraps. The colored shading and dots mark the different DnaJ subfamilies and species, respectively.

Due to the large number and low conservation of DnaJ family genes, we analyzed this family separately from the other HSP families and divided it into two groups for phylogenetic analysis. As shown in Figure 2 and Figure S2, the DnaJA and DnaJB subfamily members of *R. dominica* formed corresponding branches with homologous proteins from other species. However, the branch containing *RdDnaJ-1* is closer to some DnaJB proteins, possibly due to the high diversity of DnaJ protein sequences. Therefore, the classification of these genes needs to be further verified by conserved domain analysis. In addition, we did not observe any species-specific duplication events in the DnaJ family. Almost all branches of the DnaJ phylogenetic trees are composed of single sequences from different species, indicating a conserved evolutionary pattern among species in this family.

### 3.3. Gene Structure and Conserved Motif Analysis

**Figure 3:**
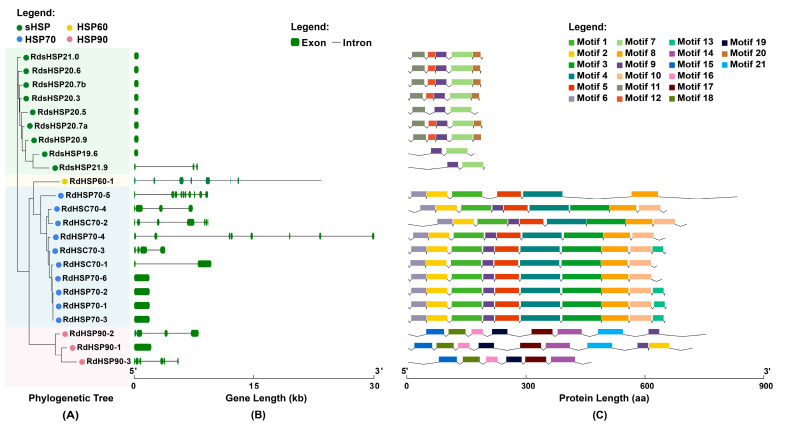
Phylogenetic relationship (**A**), gene structure (**B**), and conserved motif analysis (**C**) of HSP90, HSP70, HSP60, and sHSP family members in *Rhyzopertha dominica*. The maximum-likelihood phylogenetic tree was constructed using RAxML with 1000 bootstraps. The colored shading and dots both mark the different HSP families. The green boxes and black lines represent exon and intron regions in the gene structure, respectively. All motifs were identified using the MEME database and displayed in different colors.

**Figure 4:**
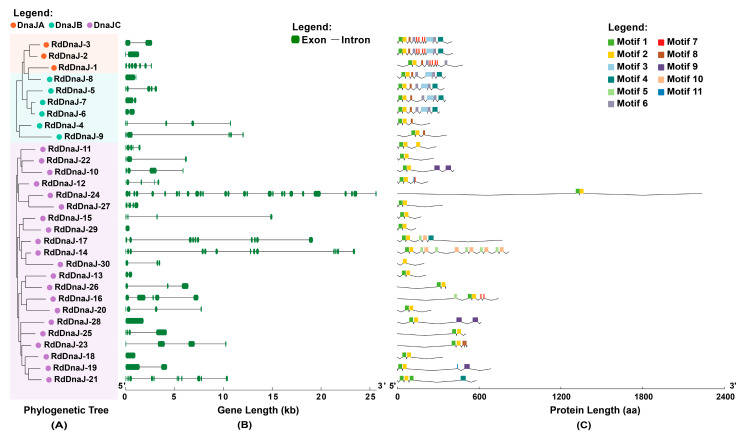
Phylogenetic relationship (**A**), gene structure (**B**), and conserved motif analysis (**C**) of DnaJ family members in *Rhyzopertha dominica*. The maximum-likelihood phylogenetic tree was constructed using RAxML with 1000 bootstraps. The colored shading and dots mark the different DnaJ subfamilies. The green boxes and black lines represent exon and intron regions in the gene structure, respectively. All motifs were identified using the MEME database and displayed in different colors.

The intraspecific phylogenetic relationships, gene structures, and conserved motif analyses of *R. dominica* HSP genes are illustrated in Figure 3 and Figure 4. As displayed in Figure 3A, all HSP sequences were grouped into different gene families consistent with their annotation results. Gene structure analysis (Figure 3B) revealed significant variation in the exon–intron composition among the HSP gene families. sHSP family members are simpler in structure, except for *RdsHSP21.9*, which has three exons, while all other genes have only one exon. The HSP70 family members show relatively greater structural diversity, with some genes, such as *RdHSP70-1*, having no introns, while others contain up to nine introns. Of the three HSP90 genes, *RdHSP90-1* consists of a single exon, whereas the other two HSP90 members have more complex gene structures. The HSP60 gene (*RdHSP60-1*) contains eight exons and has a relatively long gene length.

Distinct motif patterns across the four HSP families could be observed by performing a conserved motif analysis using the MEME suite (Figure 3C, Table S2). While the motif compositions varied significantly between families, members within the same family shared similar motif composition and arrangement. For example, in the HSP90 family, motifs 15, 18, 16, 19, 17, and 14 were consistently found in all three HSP90 genes, demonstrating a conserved functional architecture. Similarly, the HSP70 genes predominantly contained motifs 6, 2, 1, 5, 4, 3, 8, and 10. Gene structure and motif analyses indicated that although members from the same HSP family may have large differences in gene structure, they still share similar conserved structural domains and arrangement at the protein level.

In the DnaJ gene family, intraspecific clustering analysis (Figure 4A, Table S3) revealed that *RdDnaJ-1* still did not group with other DnaJA subfamily members, consistent with the results of multispecies phylogenetic analysis. Additionally, the gene structure of the DnaJ family is considerably more complex than that of the other four HSP families, as shown in Figure 4B. Among the 30 genes, only 3 consist of a single exon, while the most structurally complex gene (*RdDnaJ-24*) contains up to 27 exons.

As illustrated in Figure 4C, the motif analysis revealed that only 11 significant conserved motifs were detected among the 30 DnaJ protein sequences, indicating considerable sequence variability within the family. However, motif 1 and motif 2 were found in almost all DnaJ sequences, which represent the core regions of the J-domain and include key elements such as the conserved HPD motif. Additionally, four repeats of motif 7, which corresponds to the “CXXCXGXG” type zinc finger pattern, were identified in three DnaJA sequences including *RdDnaJ-1*. This distinctive characteristic makes it easy to distinguish DnaJA genes from the DnaJB and DnaJC genes. Furthermore, the Gly and Phe-rich region represented by motif 8 was found in all DnaJA and DnaJB genes, but was absent in DnaJC. Compared to the other two subfamilies, the positions of the J-domain in DnaJC sequences were more variable, and almost no other conserved domains were found except for the J-domain.

### 3.4. Evaluation of the Lethal Effects of Four Treatments on Rhyzopertha dominica

There have been no systematic studies of how HSP genes in stored-product insects respond to different storage stress conditions. To determine which HSP genes are involved and whether their responses differ across stress treatments, we first assessed the lethal effects of *R. dominica* populations under four pest control treatments to determine the optimal concentration, duration, or temperature for each stress condition (Tables S4–S7). The mortality rates recorded after each treatment were used to calculate the median lethal concentrations (or time, temperature) as shown in Table 3.

**Figure 5:**
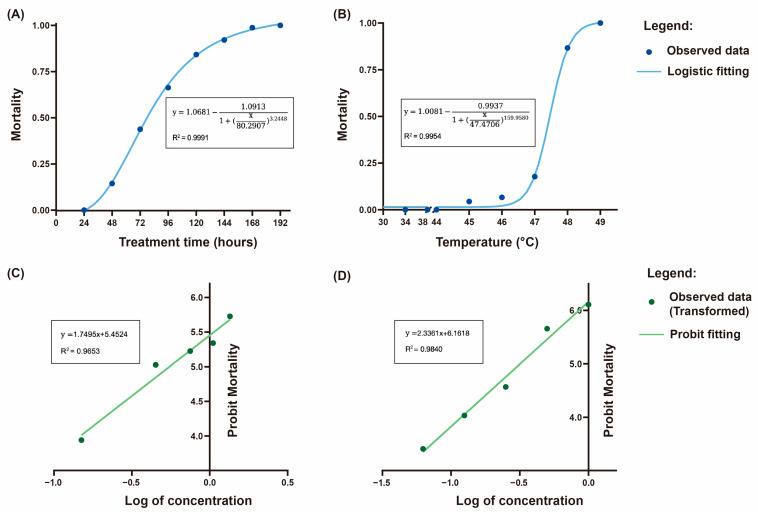
Mortality responses of *Rhyzopertha dominica* to four stress treatments based on logistic and Probit regression. The controlled nitrogen atmosphere (**A**) and extreme high temperature treatments (**B**) were fitted using logistic regression. The phosphine fumigation (**C**) and K-Obiol grain protectant treatments (**D**) were fitted using Probit regression.

For the physical treatments, we applied the logistic model to fit the observed data. The high values of *R*^2^ indicated that the fitted curves could accurately reflect the changes in mortality of test insects over time and temperature. In the controlled nitrogen atmosphere treatment with a nitrogen concentration of 99%, mortality increased steadily with exposure time (Figure 5A), from about 80 h at LT_50_ (time) to almost double at 158 h at LT_90_ (time), suggesting that the mortality increased significantly with exposure duration. The temperature stress treatments showed that *R. dominica* could tolerate a certain level of high temperature for a short period of time, but once the temperature exceeded a threshold, the mortality rate increased dramatically (Figure 5B). The narrow difference of approximately 1.3 °C between LT_10_ (Temp) and LT_90_ (Temp) suggests that even slightly exceeding the threshold led to a rapid increase in the mortality of *R. dominica*. Clarification of critical high temperature thresholds for different species is critical for effective application of heat treatments in pest management strategies.

For the chemical treatments, the Probit regression equation was used to describe the relationship between chemical concentration and pest mortality, as this model is widely used to analyze dose–response data. Both the fumigation treatment with phosphine (Figure 5C) and the grain protectant treatment with K-Obiol (Figure 5D) showed strong correlations between the log of concentration and Probit mortality. The median lethal concentrations for phosphine and K-Obiol treatments were 0.551 g/m^3^ and 0.318 μg/cm^2^, respectively. However, to ensure complete mortality of all test insects, the required concentrations would need to exceed 11.779 g/m^3^ and 3.151 μg/cm^2^ (LC_99_), respectively.

### 3.5. Transcriptome Sequencing and Expression Patterns of RdHsps

To explore the expression patterns of HSP genes under different stress conditions, we conducted transcriptome sequencing on 24 *R. dominica* adult samples exposed to the four treatments mentioned above (including control samples). All sequenced samples were categorized into four comparison groups based on the different treatments: controlled nitrogen atmosphere (CNA), extreme high temperature (EHT), phosphine fumigation (PF), and K-Obiol grain protectant (KGP). Each group consisted of three treatment samples and three control samples. The treatment samples were applied at median lethal times or concentrations (the highest temperature that did not induce coma in the insects was used in the treatment), as detailed in Table 4. The total amount of the raw bases generated from each sample ranged from 5.30 Gb to 8.49 Gb. The Q20 scores for all samples exceeded 98%, and the Q30 scores were above 94%. Additionally, the total number of clean reads of each sample exceeded 35 million, with a mapping rate of over 80%. These results confirmed the high quality and reliability of the transcriptome sequencing data.

**Figure 6:**
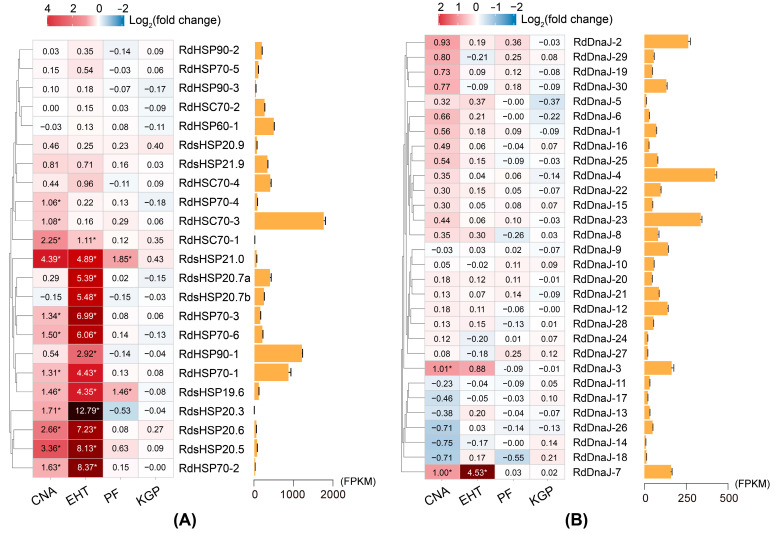
Stress-induced changes and basal levels of HSP genes expression in *Rhyzopertha dominica*. The expression patterns of HSP90, HSP70, HSP60, and sHSP genes are displayed in (**A**) and DnaJ genes in (**B**). The heat maps illustrate the log_2_(fold changes) in gene expression across the different stress treatments. Log_2_(fold change) values marked with an asterisk (*) indicate that the gene was significantly differentially expressed under the corresponding stress condition. The color of the blocks represents different levels of fold change, with red indicating upregulation and blue indicating downregulation. The deep red and black blocks in the heat maps indicate extremely high fold changes (Log2(fold change) > 4 in panel A, Log2(fold change) > 2 in panel B). The clustering pattern based on gene expression profiles is given on the left of the heatmap. The bar chart on the right shows the basal expression levels (FPKM) of HSP genes under non-stressed conditions. Each bar shows means ± standard errors.

The expression patterns of *R. dominica* HSP genes in response to the four stored-product pest control methods were analyzed and are displayed in Figure 6. Among the four treatments, extreme high temperature stress exhibited the most extensive induction of HSP genes in *R. dominica*, both in the number of genes induced and the fold change in their expression. The results showed that heat stress strongly induced the expression of multiple genes from the HSP90, HSP70, sHSP, and DnaJ families. Among them, the expression of 12 genes increased more than 10-fold, with the expression of *RdsHSP20.3*, *RdsHSP20.6*, *RdsHSP20.5*, and *RdHSP70-2* increased over 100-fold. Notably, although *RdsHSP20.3* was expressed at very low levels under non-stress conditions, it showed the highest gene induction level under heat stress, with an increase of more than 7000-fold. These findings suggest that heat stress triggers a strong and widespread activation of the HSP genes to help the insect cope with elevated temperatures.

Controlled nitrogen atmosphere treatment also resulted in upregulation of several HSP genes. A total of 14 genes were significantly increased after treatment, including seven HSP70, five sHSP, and two DnaJ genes. The upregulation of most of the genes was not as strong as that of high temperature stress, and most of the induced genes were from the HSP70 family. However, some genes, such as *RdHSP70-4*, *RdHSC70-3*, and *RdHSC70-1*, exhibited a stronger response under controlled nitrogen atmosphere stress than under the heat stress, suggesting their specific role in adaptation to low-oxygen, high-nitrogen conditions.

The chemical stress treatments, including phosphine fumigation and K-Obiol grain protectant, had significantly less effect on HSP gene expression compared to the physical stress treatments. Under phosphine fumigation, only *RdsHSP19.6* and *RdsHSP21.0* were significantly induced, and the overall response of HSP genes was weaker than that observed under controlled nitrogen atmosphere or extreme high temperature treatment. The K-Obiol grain protectant treatment had the least effect on the induction of HSP genes, with no significant changes observed in the expression of any HSP genes. These results suggest that these chemical treatments do not strongly activate the heat shock protein response in *R. dominica*.

Among all gene families, stress treatments induced the most significant induction in the HSP70 and sHSP families. Most of the genes in these families showed significant upregulation under at least one stress condition, especially under extreme heat treatment conditions, highlighting their critical role in coping with negative environmental stress. Only one gene (*RdHSP90-1*) from the HSP90 family was upregulated under stress, but the intensity of its response was relatively modest compared to that of the HSP70 and sHSP genes. Moreover, the only HSP60 gene in *R. dominica* also showed stable expression in all four treatments, with no significant response to any stress conditions. Of the 30 DnaJ genes, only *RdDnaJ-7* exhibited significant induction with a log_2_(fold change) of 4.53 under heat stress, whereas most of the other DnaJ genes showed little change in all treatments. As HSP70 gene co-chaperones, the expression patterns of DnaJ genes were not entirely consistent with that of the HSP70 genes, suggesting that the interaction between DnaJ and HSP70 may not be a simple 1:1 relationship, but rather a more complex mechanism of interaction.

**Figure 7:**
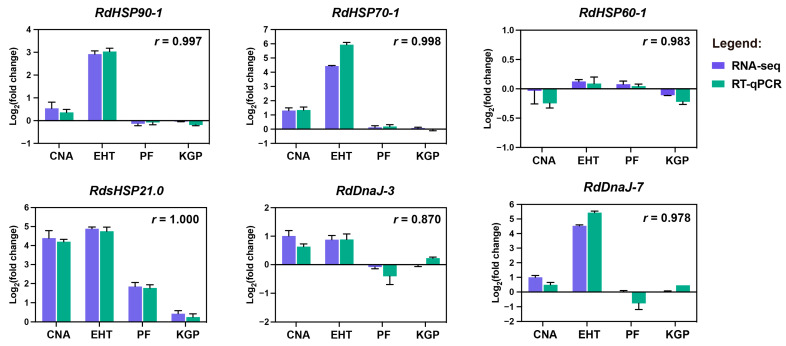
RT-qPCR validation of transcriptome analysis results of six HSP genes under different treatments in *Rhyzopertha dominica*. Different treatments included controlled nitrogen atmosphere (CNA), extreme high temperature (EHT), phosphine fumigation (PF), and K-Obiol grain protectant (KGP). Pearson correlation coefficients (*r*) between RNA-Seq and RT-qPCR data are shown for each gene. *RdEF1α* was used as the reference gene for RT-qPCR validation.

RT-qPCR was performed to further validate the expression patterns of HSP genes identified through transcriptome analysis in *R. dominica*. Six genes (*RdHSP90-1*, *RdHSP70-1*, *RdHSP60-1*, *RdHSP21.0*, *RdDnaJ-3*, and *RdDnaJ-7*) from five gene families were selected for verification. As shown in Figure 7 and Figure S3, most of the selected genes exhibited consistent expression trends and high Pearson correlation coefficients (*r*) between the transcriptome and RT-qPCR results, validating the overall reliability of the RNA-Seq data. Although some inconsistencies were observed between the RNA-Seq and RT-qPCR results for specific genes, such as *RdDnaJ-3* under KGP treatment, these discrepancies were minor (within one-fold) and likely due to technical variations, primer efficiency, or small gene expression changes, which were considered acceptable.

## 4. Discussion

In this study, we performed a comprehensive genome-wide analysis of the HSP gene superfamily in the *R. dominica* genome, identifying 53 HSP genes from five families: HSP90, HSP70, HSP60, sHSP, and DnaJ. The overall distribution and characteristics of these HSP families were consistent with previously reported findings, indicating their evolutionary conservation across taxa [46,71]. Among the five HSP families in *R. dominica*, DnaJ family is the largest with 30 members, exhibiting considerable variation in physiological and biochemical characteristics, which is consistent with the findings in *N. lugens* and *B. mori* [15,61]. Phylogenetic analysis further confirmed the conservation of HSP families across species. However, there was higher variability in branch lengths and species-specific expansion in the small HSP family, likely due to its role in responding to diverse stress conditions across species, which contributes to its rapid evolutionary divergence [72,73].

Gene structure and protein motif analyses revealed clear distinctions among the HSP gene families in *R. dominica*. The sHSP genes exhibited simple gene structures, with most members only containing a single exon, which may confer an advantage in rapid and high-level transcriptional responses to stress [74]. This has been proposed as a general feature of sHSP genes in insects [24,75], where rapid activation is crucial for thermotolerance. Interestingly, this pattern was also observed in many stress-induced HSP genes from other families. The conservation of specific motifs within each family further emphasizes the functional importance of these domains. For instance, the J-domain found in almost all DnaJ proteins is essential for its role in assisting HSP70 chaperone functions, while the presence of zinc finger motifs in some DnaJ proteins suggests additional roles in transcriptional regulation or protein stabilization [29]. These findings highlight the evolutionary pressures that have shaped the HSP gene families to maintain core functions, while also allowing for specialization in response to distinct stress environments.

Among the four treatments, the extreme heat stress was the most potent inducer of HSP gene expression, particularly within the sHSP family, which exhibited the largest fold increases. This is consistent with the well-established role of HSPs in mediating thermotolerance. The nitrogen atmosphere treatment, which created a hypoxic (low-oxygen) environment [76], also resulted in the induction of several HSP genes, particularly those from the HSP70 family. Certain HSP70 genes were specifically induced under controlled atmosphere stress, suggesting their specific roles in hypoxic environment adaptation. The important role of HSP70 genes in hypoxia adaptation was also reported in *Sarcophaga crassipalpis* [77] and *Bactrocera dorsalis* [78].

In contrast, chemical treatments led to relatively weak induction of HSP genes. Phosphine acts by inhibiting the mitochondrial complex, leading to ROS accumulation, which causes oxidative stress and cellular damage in insects [79,80,81]. Only two genes (*RdsHSP19.6* and *RdsHSP21.0*) showed moderate upregulation in response to phosphine treatment, likely due to its primary mode of action in disrupting cellular metabolism, particularly mitochondrial function, rather than directly causing protein denaturation. As the active ingredient of K-Obiol grain protectant, deltamethrin functions as a neurotoxin by interfering with sodium channels, and it hardly caused any obvious changes in HSP gene expression in *R. dominica*, which is consistent with findings in *Meligethes aeneus* [82]. However, in *Monochamus alternatus* [83] and *Liposcelis bostrychophila* [71], deltamethrin exposure induced the expression of certain HSP genes, although not a large-scale response. These results contrast with the strong HSP response triggered by heat stress, where direct protein damage requires more extensive activation of heat shock proteins to refold and degrade damaged proteins. Related evidence can also be obtained from the GO analysis (Figures S4–S7), where heat stress led to significant enrichment of terms associated with protein folding, peptide metabolism, and peptide biosynthesis compared to the other stresses, suggesting widespread protein damage.

The distinct expression patterns among the HSP families further illustrate their roles in stress adaptation. Multiple HSP70 and sHSP genes exhibited significant upregulation under stresses, highlighting their key role in maintaining protein homeostasis by refolding misfolded proteins and preventing aggregation under stress, as seen in other species [84,85,86,87]. However, the expressions of HSP90 and HSP60 genes were relatively stable, probably due to their primary role in housekeeping functions, such as protein folding, chaperoning newly synthesized proteins, and supporting mitochondrial activity under non-stress conditions [20,88]. To our surprise, as co-chaperones of HSP70, only two of the 30 DnaJ genes were significantly induced after stress, contrasting sharply with the robust induction of HSP70 genes. A similar response was also observed in *N. lugens*, in which further biological function research indicated that although DnaJ proteins are co-chaperones of HSP70, they still have various functions independent of HSP70 [15]. Despite significant progress in the study of HSP70 and DNAJ interactions in humans and model organisms, there is still a major gap in insect research, which requires further investigation.

In summary, this study was the first comprehensive analysis of the HSP gene superfamily in *R. dominica*. A total of 53 HSP genes were identified and their evolutionary relationships, gene structures, conserved domains, and expression patterns under four storage-related stress conditions were analyzed, revealing the potential roles of different HSP genes in response to diverse stressors. Based on these findings, future research could further explore the biological functions of these HSP genes and investigate the correlations between key genes and stress tolerance, which could provide molecular targets and biomarkers for the development of precision control technologies and resistance monitoring methods, thereby contributing to the advancement of more efficient and sustainable pest management strategies for stored products.

## Figures and Tables

**Table 1:** Information of HSP90, HSP70, HSP60, and sHSP family genes in *Rhyzopertha dominica*.

Family	Gene Name	Scaffold	Strand	Protein Length (aa)	Molecular Weight (kDa)	PI	Subcellular Location
HSP90	*RdHSP90-1*	QCXZ01000042.1	−	718	82.3	5.01	Cytoplasmic
	*RdHSP90-2*	QCXZ01000138.1	+	752	86.1	4.90	Cytoplasmic/Extracellular
	*RdHSP90-3* *(partial)*	QCXZ01000137.1	+	471	-	-	-
HSP70	*RdHSP70-1*	QCXZ01000003.1	+	652	71.1	5.43	Cytoplasmic
	*RdHSP70-2*	QCXZ01000003.1	−	649	70.8	5.44	Cytoplasmic
	*RdHSP70-3*	QCXZ01000003.1	−	649	70.7	5.44	Cytoplasmic
	*RdHSP70-4*	QCXZ01000003.1	+	649	71.4	5.28	Cytoplasmic
	*RdHSP70-5*	QCXZ01000041.1	−	831	93.0	5.69	Nuclear
	*RdHSP70-6*	QCXZ01000053.1	+	641	70.4	5.82	Cytoplasmic
	*RdHSC70-1*	QCXZ01000137.1	+	629	69.1	5.45	Cytoplasmic
	*RdHSC70-2*	QCXZ01000137.1	−	703	77.0	5.88	Mitochondrial
	*RdHSC70-3*	QCXZ01000137.1	−	650	71.1	5.33	Cytoplasmic
	*RdHSC70-4*	QCXZ01000137.1	−	653	72.8	5.17	Endoplasmic Reticulum
HSP60	*RdHSP60-1*	QCXZ01000001.1	−	591	63.4	5.52	Mitochondrial
sHSP	*RdsHSP21.0*	QCXZ01000001.1	+	191	21.0	5.62	Mitochondrial/Nuclear/Extracellular
	*RdsHSP20.6*	QCXZ01000001.1	+	186	20.6	6.85	Cytoplasmic/Mitochondrial
	*RdsHSP20.5*	QCXZ01000001.1	+	178	20.5	6.52	Nuclear/Cytoplasmic
	*RdsHSP19.6*	QCXZ01000003.1	−	167	19.6	6.14	Nuclear/Cytoplasmic
	*RdsHSP20.7a*	QCXZ01000041.1	+	186	20.7	5.86	Extracellular/Cytoplasmic/Nuclear
	*RdsHSP20.7b*	QCXZ01000064.1	−	183	20.7	6.60	Mitochondrial/Cytoplasmic
	*RdsHSP20.9*	QCXZ01000064.1	−	183	20.9	6.52	Nuclear
	*RdsHSP20.3*	QCXZ01000064.1	+	179	20.3	7.03	Extracellular
	*RdsHSP21.9*	QCXZ01000137.1	−	192	21.9	5.61	Nuclear

**Table 2:** Information of DnaJ family genes in *Rhyzopertha dominica*.

Subfamily	Gene Name	Scaffold	Strand	Protein Length (aa)	Molecular Weight (kDa)	PI	Subcellular Location
DnaJA	*RdDnaJ-1*	QCXZ01000041.1	+	481	52.9	9.29	Mitochondrial
	*RdDnaJ-2*	QCXZ01000042.1	−	407	45.5	6.29	Nuclear
	*RdDnaJ-3*	QCXZ01000137.1	−	401	45.0	6.44	Nuclear
DnaJB	*RdDnaJ-4*	QCXZ01000138.1	+	239	27.3	9.20	Nuclear
	*RdDnaJ-5*	QCXZ01000042.1	−	344	38.9	5.88	Cytoplasmic
	*RdDnaJ-6*	QCXZ01000138.1	−	310	35.0	8.77	Cytoplasmic
	*RdDnaJ-7*	QCXZ01000086.1	+	354	38.9	9.23	Cytoplasmic
	*RdDnaJ-8*	QCXZ01000001.1	−	356	40.3	5.66	Nuclear
	*RdDnaJ-9*	QCXZ01000003.1	−	362	41.7	8.88	Nuclear
DnaJC	*RdDnaJ-10*	QCXZ01000064.1	−	416	48.1	8.10	Nuclear
	*RdDnaJ-11*	QCXZ01000003.1	+	285	33.0	7.67	Nuclear
	*RdDnaJ-12*	QCXZ01000075.1	−	224	24.6	7.45	Extracellular
	*RdDnaJ-13*	QCXZ01000075.1	+	210	24.2	9.52	Mitochondrial
	*RdDnaJ-14*	QCXZ01000137.1	−	818	94.0	5.91	Cytoplasmic
	*RdDnaJ-15*	QCXZ01000137.1	+	175	19.5	6.97	Nuclear
	*RdDnaJ-16*	QCXZ01000138.1	−	743	84.8	9.46	Nuclear
	*RdDnaJ-17*	QCXZ01000138.1	−	771	89.8	8.78	Plasma Membrane
	*RdDnaJ-18*	QCXZ01000137.1	+	335	40.5	8.74	Cytoplasmic
	*RdDnaJ-19*	QCXZ01000064.1	+	687	80.0	8.00	Nuclear
	*RdDnaJ-20*	QCXZ01000137.1	−	247	29.2	9.24	Nuclear
	*RdDnaJ-21*	QCXZ01000001.1	−	580	65.7	7.66	Nuclear/Cytoplasmic/Mitochondrial
	*RdDnaJ-22*	QCXZ01000137.1	−	265	31.8	8.47	Nuclear
	*RdDnaJ-23*	QCXZ01000001.1	−	512	58.5	8.44	Nuclear
	*RdDnaJ-24*	QCXZ01000064.1	−	2238	254.7	6.78	Nuclear
	*RdDnaJ-25*	QCXZ01000042.1	−	503	58.1	5.95	Cytoplasmic
	*RdDnaJ-26*	QCXZ01000003.1	−	356	41.7	8.96	Plasma Membrane
	*RdDnaJ-27*	QCXZ01000053.1	+	332	38.9	8.57	Cytoplasmic
	*RdDnaJ-28*	QCXZ01000064.1	−	615	71.2	8.84	Nuclear
	*RdDnaJ-29*	QCXZ01000041.1	−	136	15.9	4.99	Nuclear
	*RdDnaJ-30*	QCXZ01000041.1	+	196	22.8	9.39	Mitochondrial/Plasma Membrane

**Table 3:** Lethal effects and fitting information of four treatments on *Rhyzopertha dominica*.

Treatment	LC/LT_10_	LC/LT_50_	LC/LT_90_	Fit Model	R^2^
Controlled Nitrogen Atmosphere	40.792 h	80.291 h	158.035 h	Logistic	0.999
Extreme High Temperature	46.823 °C	47.471 °C	48.127 °C	Logistic	0.995
Phosphine Fumigation	0.102 g/m^3^	0.551 g/m^3^	2.978 g/m^3^	Probit	0.965
K-Obiol Grain Protectant	0.090 μg/cm^2^	0.318 μg/cm^2^	1.125 μg/cm^2^	Probit	0.984

**Table 4:** Summary of transcriptome sequencing data of *Rhyzopertha dominica* in this study.

Group Name	Sample Name	Treatment Details	Raw Bases	Q20 (%)	Q30 (%)	Clean Reads	Total Mapping Reads (Rates)
CNA	N1	Controlled nitrogen atmosphere treatment (99% N_2_ for 80 h)	8.41 Gb	98.42	95.35	52,279,194	43,583,325 (83.37%)
	N2		6.94 Gb	98.73	96.15	43,780,126	36,996,673 (84.51%)
	N3		7.59 Gb	98.36	95.14	48,838,362	41,920,620 (85.84%)
	CN1	Control for controlled nitrogen atmosphere treatment (78% N_2_ for 80 h)	7.24 Gb	98.55	95.65	46,305,876	38,547,717 (83.25%)
	CN2		7.20 Gb	98.55	95.68	44,932,154	37,046,999 (82.45%)
	CN3		8.02 Gb	98.26	94.97	52,145,254	41,805,673 (80.17%)
EHT	H1	Extreme high temperature treatment (46 °C for 3 h)	6.89 Gb	98.98	96.95	41,938,936	34,799,525 (82.98%)
	H2		5.98 Gb	98.93	96.76	36,007,342	30,014,415 (83.36%)
	H3		6.49 Gb	98.98	96.89	42,156,452	34,320,294 (81.41%)
	CH1	Control for extreme high temperature treatment (30 °C for 3 h)	6.66 Gb	98.91	96.74	40,544,948	34,467,807 (85.01%)
	CH2		6.61 Gb	98.92	96.75	40,519,056	34,437,334 (84.99%)
	CH3		5.30 Gb	98.91	96.69	35,313,172	29,893,814 (84.65%)
PF	P1	Phosphine fumigation treatment (0.55 g/m^3^ of phosphine gas for 20 h)	8.49 Gb	98.42	95.37	54,428,094	45,038,584 (82.75%)
	P2		7.48 Gb	98.25	94.88	47,541,036	39,146,791 (82.34%)
	P3		7.87 Gb	98.26	94.96	50,162,810	40,906,312 (81.55%)
	CP1	Control for phosphine fumigation treatment (0 g/m^3^ of phosphine gas for 20 h)	7.26 Gb	98.37	95.13	46,007,646	37,299,197 (81.07%)
	CP2		6.99 Gb	98.54	95.64	43,525,938	35,446,750 (81.44%)
	CP3		6.45 Gb	98.29	94.98	40,529,910	34,389,670 (84.85%)
KGP	K1	K-Obiol grain protectant treatment (0.32 μg/cm^2^ of deltamethrin for 24 h)	7.11 Gb	98.20	94.68	46,005,272	39,206,013 (85.22%)
	K2		6.90 Gb	98.36	95.16	44,714,288	37,767,301 (84.46%)
	K3		6.90 Gb	98.29	95.00	44,494,096	38,208,768 (85.87%)
	CK1	Control for K-Obiol grain protectant treatment (0 μg/cm^2^ of deltamethrin for 24 h)	7.56 Gb	98.38	95.19	48,579,578	40,769,074 (83.92%)
	CK2		6.98 Gb	98.27	94.88	44,928,050	37,932,915 (84.43%)
	CK3		6.88 Gb	98.29	94.97	43,128,218	36,892,070 (85.54%)

## Data Availability

Transcriptome data were deposited in the Sequence Read Archive (SRA) repository of NCBI (BioProject number: PRJNA1172111). All bioassay data and identified heat shock protein sequences can be found in Supplementary Information files.

## Supplementary Materials

The following supporting information can be downloaded at: https://www.mdpi.com/article/10.3390/insects16020127/s1, Figure S1: Phylogenetic analysis of HSP90, HSP70, HSP60, and sHSP family members from *Rhyzopertha dominica* and other species (with bootstrap values shown); Figure S2: Phylogenetic analysis of DnaJ family members from *Rhyzopertha dominica* and other species (with bootstrap values shown); Figure S3: RT-qPCR validation of transcriptome analysis results of six HSP genes under different treatments in *Rhyzopertha dominica* (*RdRPS6* as reference gene). FigureS4: GO enrichment analysis of significantly upregulated genes under controlled nitrogen atmosphere (CNA). FigureS5: GO enrichment analysis of significantly upregulated genes under extreme high treatment (EHT). Figure S6: GO enrichment analysis of significantly upregulated genes under phosphine fumigation (PF). Figure S7: GO enrichment analysis of significantly upregulated genes under K-Obiol grain protectant (KGP). Table S1: Primers used in RT-qPCR assays. Table S2: Information of conserved motifs of HSP90, HSP70, HSP60, and sHSP sequences in *Rhyzopertha dominica* searched by MEME Suite; Table S3: Information of conserved motifs of DnaJ sequences in *Rhyzopertha dominica* searched by MEME Suite; Table S4: Details of bioassay data from the extreme high temperature treatment; Table S5: Details of bioassay data from the controlled nitrogen atmosphere treatment; Table S6: Details of bioassay data from the phosphine fumigation treatment. Table S7: Details of bioassay data from the K-Obiol grain protectant treatment. Data S1: Heat shock protein sequences of *Rhyzopertha dominica*. Data S2: Heat shock protein sequences of other species used in this study.
