# Supplementary material for: Genome-Wide Identification and Characterization of Heat Shock Proteins in the Stored-Product Pest Rhyzopertha dominica (Fabricius): Phylogenetic, Structural, and Stress-Induced Expression Analyses
Source: Insects. 2025 Jan 28;16(2):127. doi: 10.3390/insects16020127 (PMC11855361; doi:10.3390/insects16020127)
Supplement: Supplementary file 1 [file insects-16-00127-s001.zip › Supplementary Figures and Tables.pdf]

Supplemental Information for:

Genome-Wide Identification and Characterization of Heat Shock Proteins in the Stored-Product Pest *Rhyzopertha dominica* (Fabricius): Phylogenetic, Structural, and Stress-Induced Expression Analyses

Yueliang Bai [1,2,*], Yanzhu Xie [2], Junji Yao [2], Fangfang Zeng [2] and Dianxuan Wang [2]

Table of contents:

**Title and authors**	**Page1**
**Figure S1**	**Page 2**
**Figure S2**	**Page 3**
**Figure S3**	**Page 4**
**Figure S4**	**Page 5**
**Figure S5**	**Page 6**
**Figure S6**	**Page 7**
**Figure S7**	**Page 8**
**Table S1**	**Page 9**
**Table S2**	**Page 10**
**Table S3**	**Page 11**
**Table S4**	**Page 12**
**Table S5**	**Page 12**
**Table S6**	**Page 13**
**Table S7**	**Page 13**

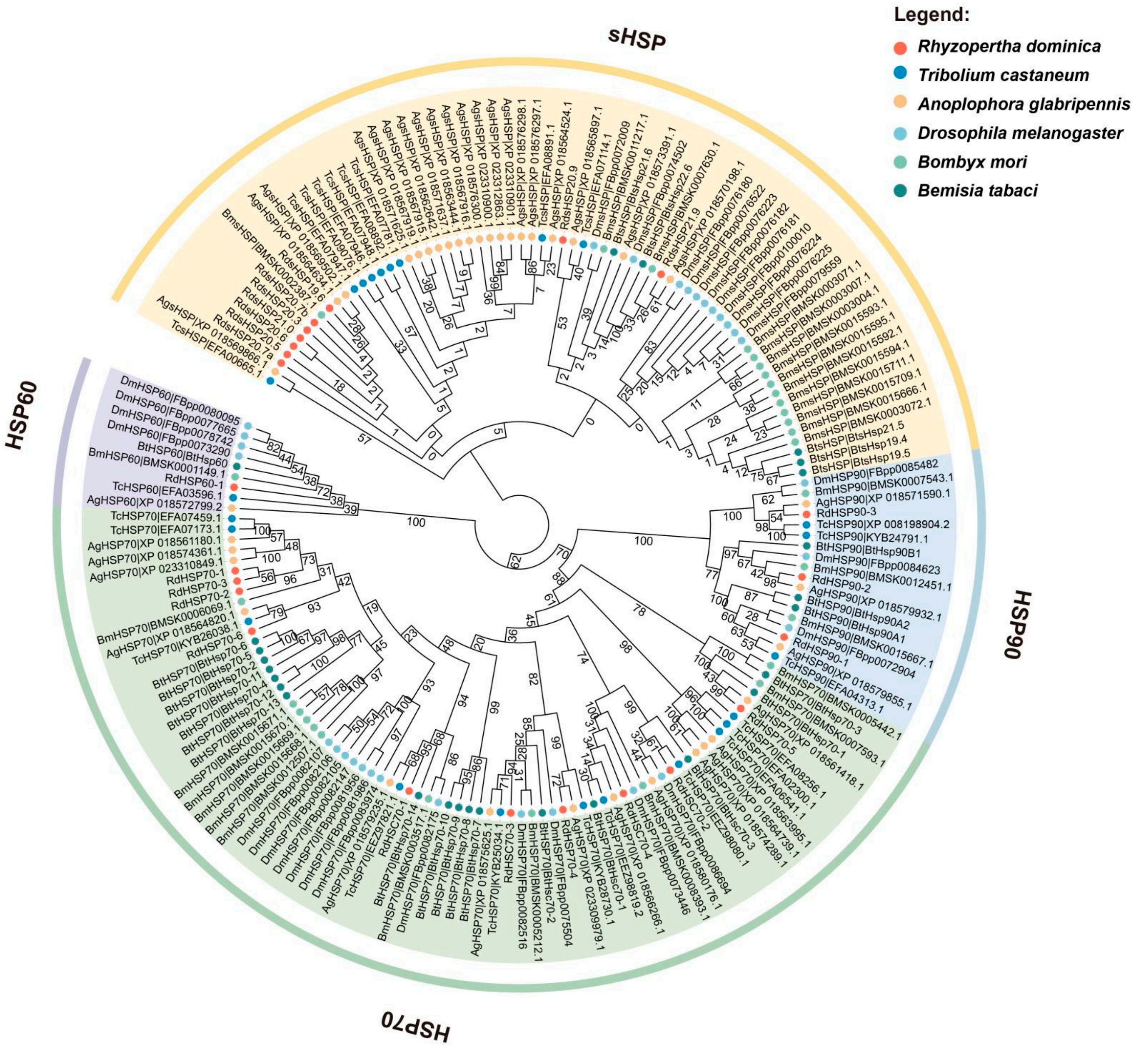

Supplementary Figure S1: Phylogenetic analysis of HSP90, HSP70, HSP60, and sHSP family members from *Rhyzopertha dominica* and other species (with bootstrap values shown). The maximum-likelihood phylogenetic tree was constructed using RAxML with 1000 bootstraps. The colored shading and dots mark the different DnaJ subfamilies and species, respectively. Numbers on branches are bootstrap portions from 1000 replicates.

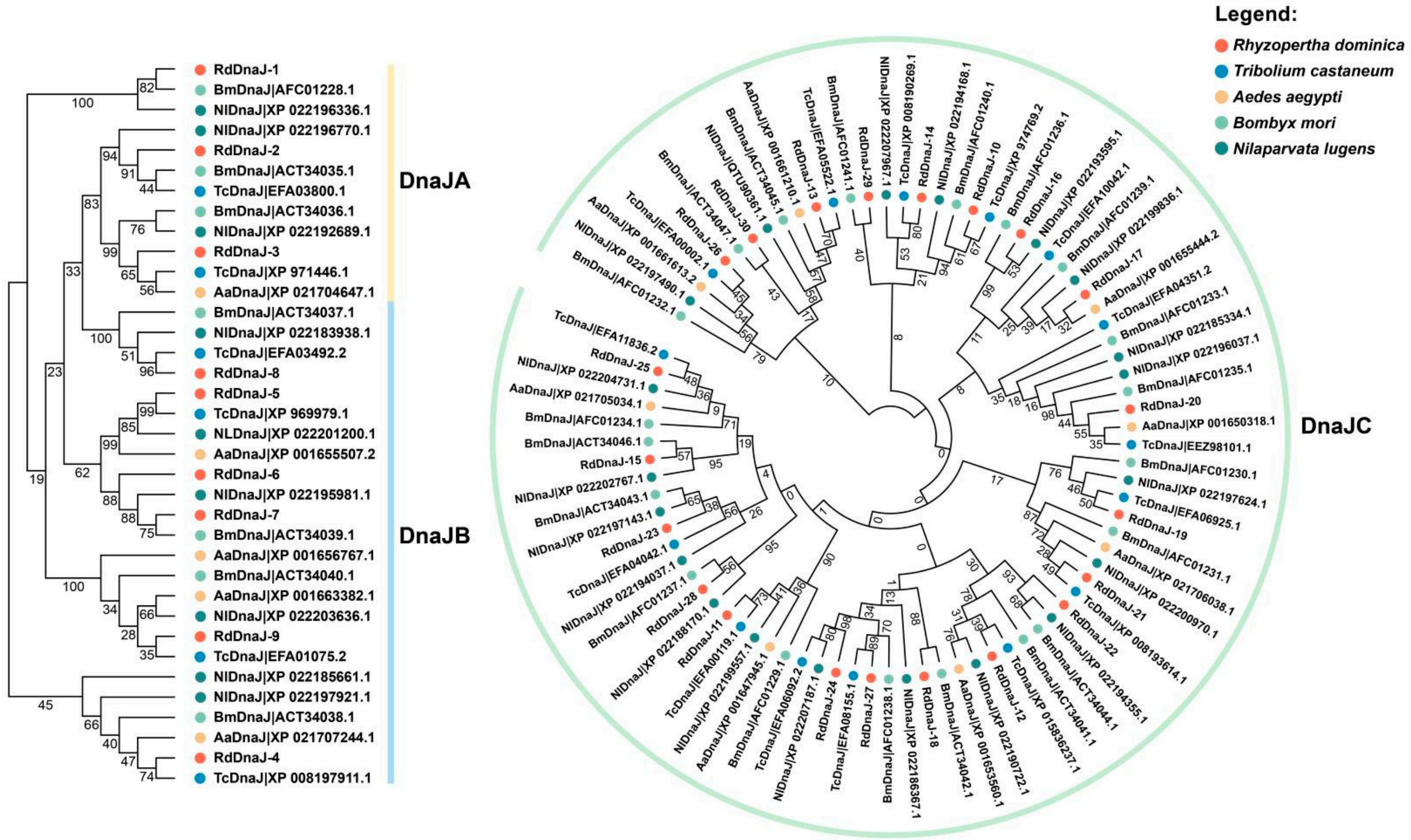

Supplementary Figure S2: Phylogenetic analysis of DnaJ family members from *Rhyzopertha dominica* and other species (with bootstrap values shown). The maximum-likelihood phylogenetic tree was constructed using RAxML with 1000 bootstraps. The colored shading and dots mark the different DnaJ subfamilies and species, respectively. Numbers on branches are bootstrap portions from 1000 replicates.

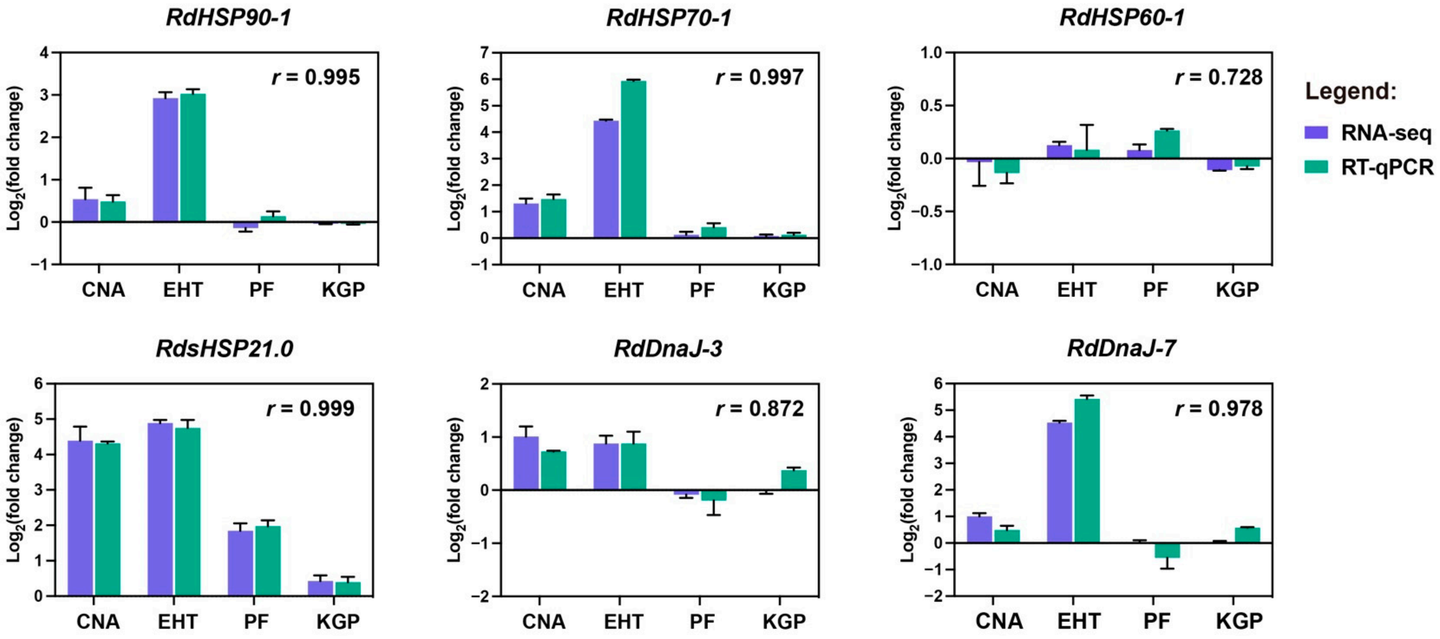

Supplementary Figure S3: RT-qPCR validation of transcriptome analysis results of six HSP genes under different treatments in *Rhyzopertha dominica*. Different treatments including controlled nitrogen atmosphere (CNA), extreme high temperature (EHT), phosphine fumigation (PF), and K-Obiol grain protectant (KGP). Pearson correlation coefficients (*r*) between RNA-Seq and RT-qPCR data are shown for each gene. *RdRPS6* was used as the reference gene for RT-qPCR validation.

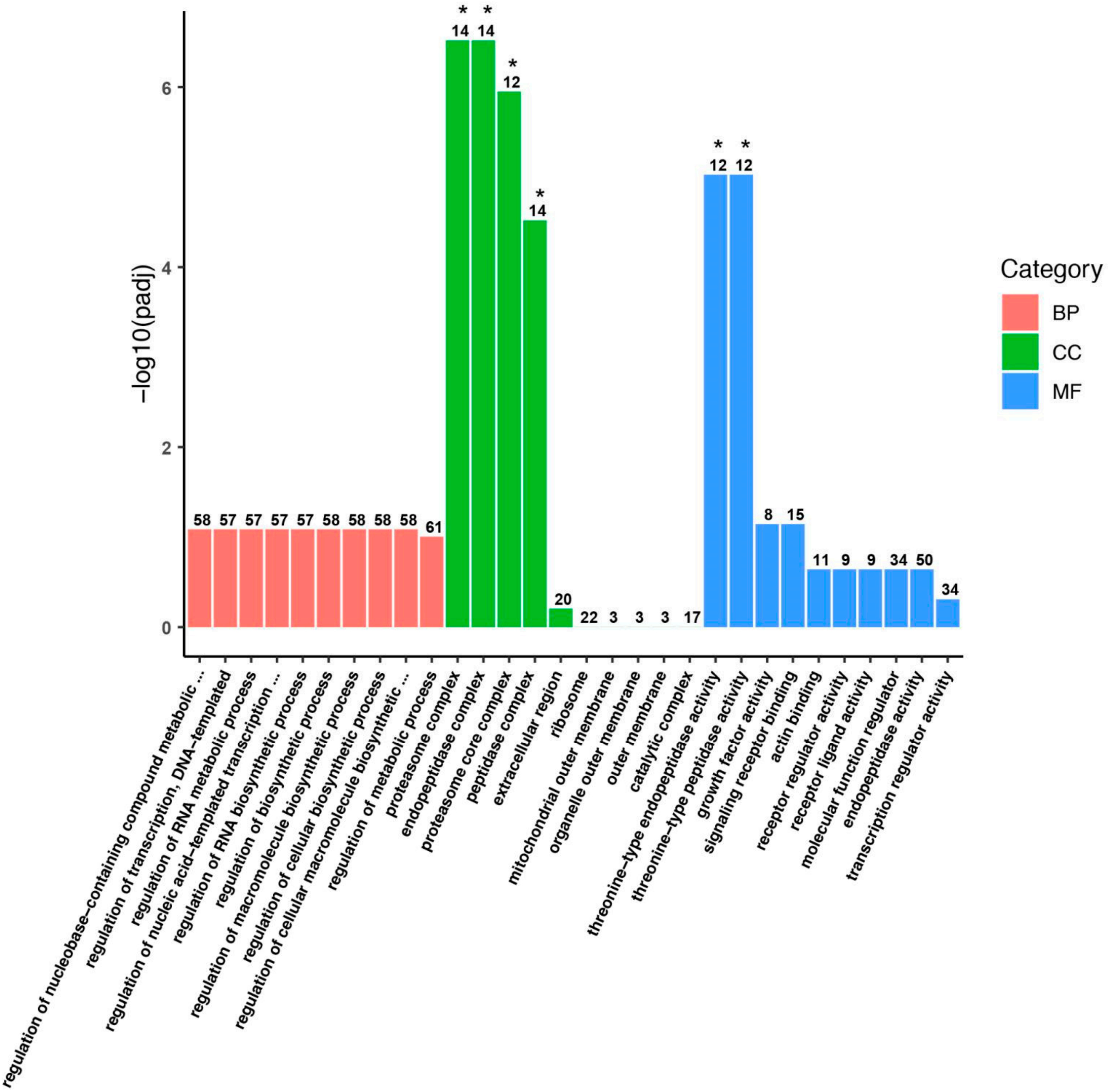

Supplementary Figure S4. GO enrichment analysis of significantly up-regulated genes under controlled nitrogen atmosphere (CNA). The ***X***-axis indicates the classifications of GO terms, including BP (Biological Process), CC (Cellular Component), and MF (Molecular Function). The ***Y***-axis represents the significance level of GO Term enrichment, indicated by -log10(padj). The top ten enriched terms form each classification are displayed. * indicates significantly enriched GO terms, with padj < 0.05.

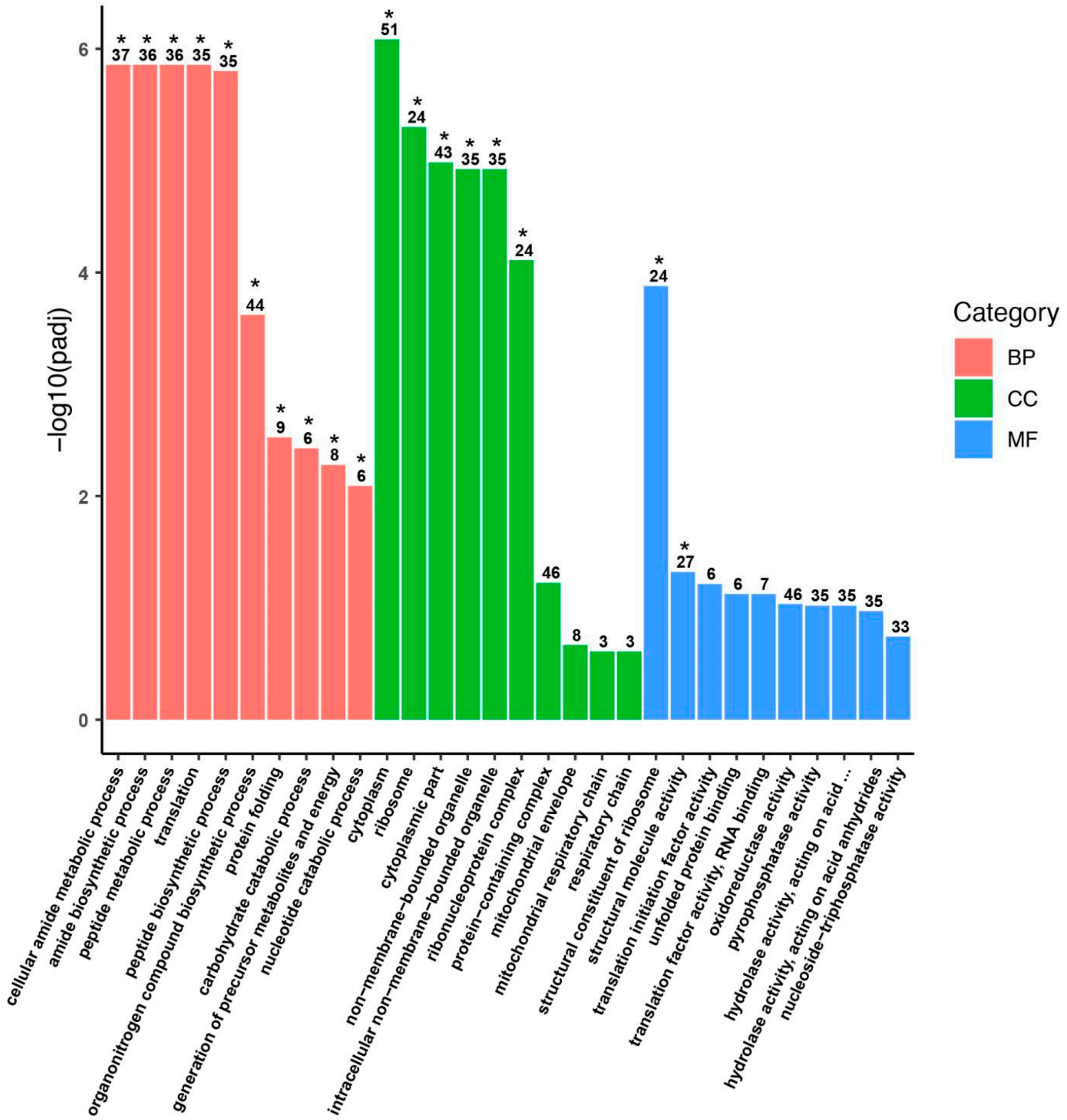

Supplementary Figure S5. GO enrichment analysis of significantly up-regulated genes under extreme high treatment (EHT). The ***X***-axis indicates the classifications of GO terms, including BP (Biological Process), CC (Cellular Component), and MF (Molecular Function). The ***Y***-axis represents the significance level of GO Term enrichment, indicated by -log10(padj). The top ten enriched terms form each classification are displayed. * indicates significantly enriched GO terms, with padj < 0.05.

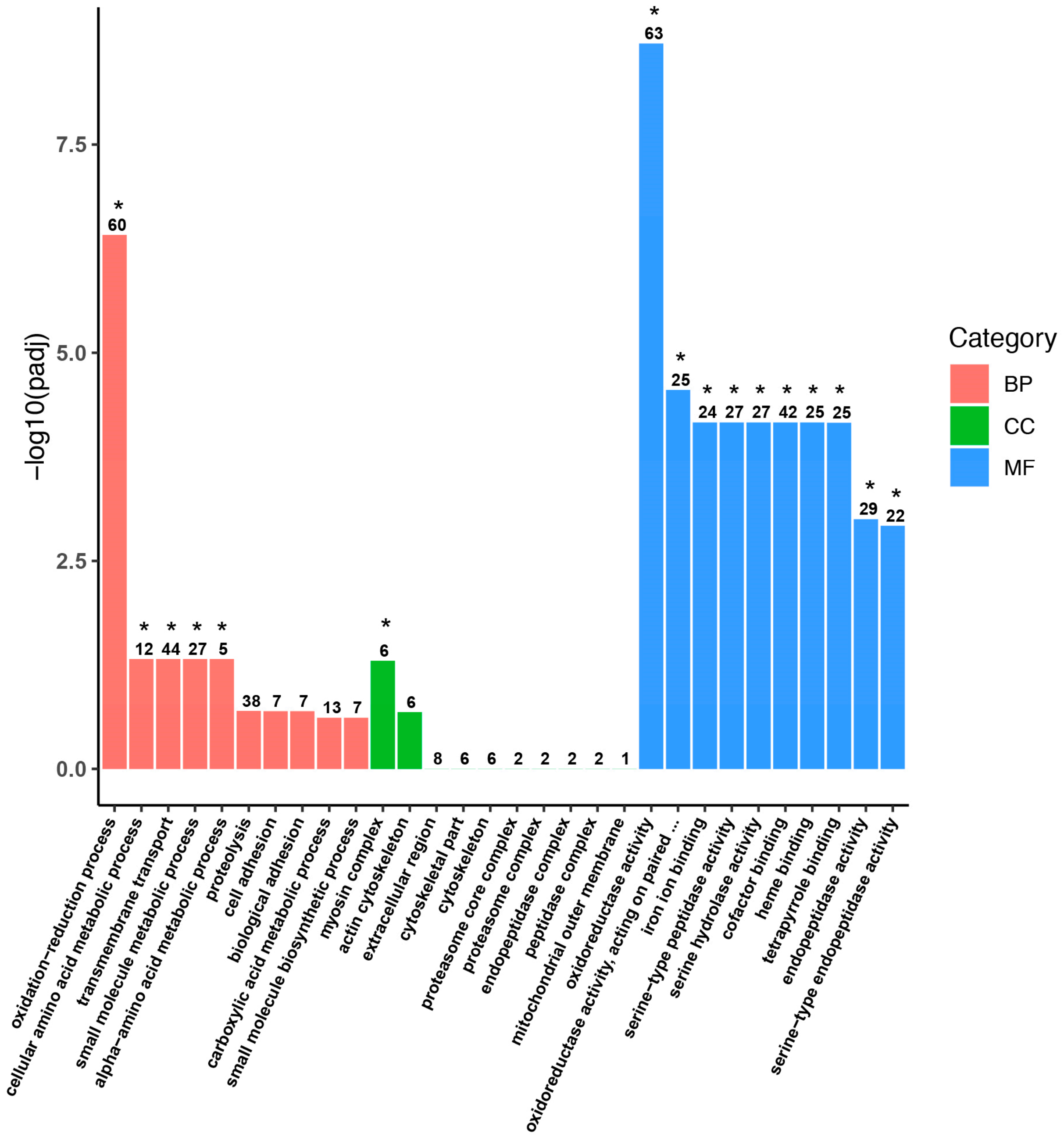

Supplementary Figure S6. GO enrichment analysis of significantly up-regulated genes under phosphine fumigation (PF). The ***X***-axis indicates the classifications of GO terms, including BP (Biological Process), CC (Cellular Component), and MF (Molecular Function). The ***Y***-axis represents the significance level of GO Term enrichment, indicated by -log10(padj). The top ten enriched terms form each classification are displayed. * indicates significantly enriched GO terms, with padj < 0.05.

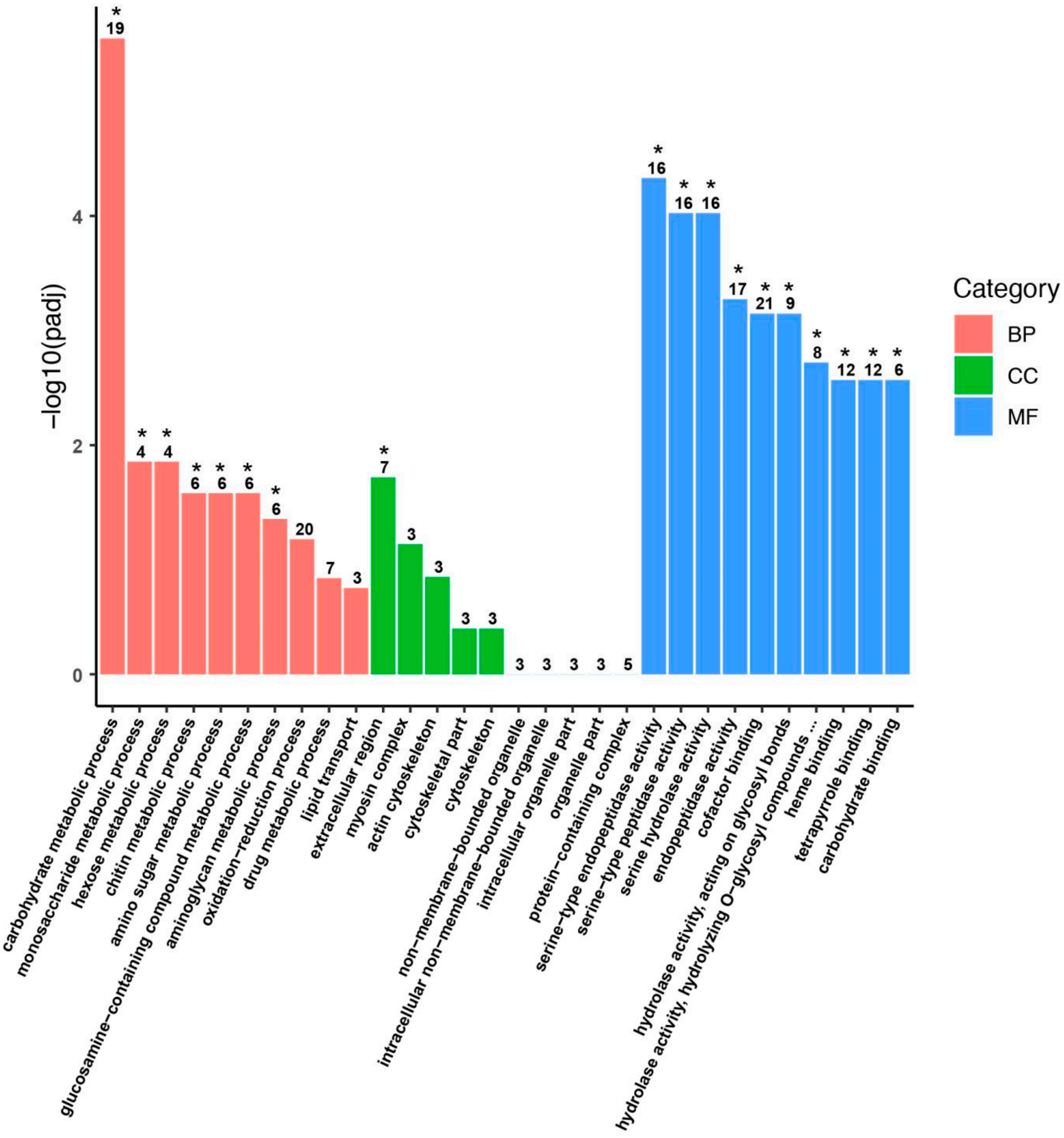

Supplementary Figure S7. GO enrichment analysis of significantly up-regulated genes under K-Obiol grain protectant (KGP). The ***X***-axis indicates the classifications of GO terms, including BP (Biological Process), CC (Cellular Component), and MF (Molecular Function). The ***Y***-axis represents the significance level of GO Term enrichment, indicated by -log10(padj). The top ten enriched terms form each classification are displayed. * indicates significantly enriched GO terms, with $padj < 0.05$.

Supplementary Table S1: Primers used in RT-qPCR assays.

Gene name	Direction	Sequence (5'-3')	Product (bp)
RdHSP90-1	F	ACAAGTCCCTCACCAACGAC	173
	R	GATAAACACCCTGCGGACAT	
RdHSP70-1	F	GAATACAAGGGCGAAACCAA	151
	R	GACGCTGTGAGTCGTTGAAA	
RdHSP60-1	F	AAGCACCAGGTTTTGGTGAC	149
	R	GATCACGATTTCGCCAACTT	
RdsHSP21.0	F	GGTTCGACAGTCAGCACAGA	142
	R	GCTCGTCTGGTTTCTCTTCG	
RdDnaJ-3	F	GGATGTTCCGCTGATGATTT	179
	R	CCTTCTTTCAATGCCTGCTC	
RdDnaJ-7	F	TTCCAGCACTTCTTCGACCT	169
	R	GGCTACCGTGGAAGTTGAAA	
RdEF1α	F	GGTCAGATTTCCAACGGCTA	162
	R	GAGGTTTACGATGGCAGCAT	
RdRPS6	F	ATGGTGAACGCAAAAGGAAG	163
	R	TTGCGAATTTTGGAAGCTCT	

Supplementary Table S2: Information of conserved motifs of HSP90, HSP70, HSP60, and sHSP sequences in *Rhyzopertha dominica* searched by MEME Suite.

Motif ID	Sequence	Length (aa)
Motif 1	GETKTFAPEEISSMVLTKMKETAEAYLGCKVKDAVITVPAYFNDS QRQATKDAGTIAGLNVLRIINEPTAAALAYGL	77
Motif 2	DTERLIGDAAKNQVAMNPSNTVFDAKRLIGRKFDDPKIQQDMK HWPFKVVNDG	53
Motif 3	IQDVLLVDVTPLSLGIETAGGVMTKJIERNARIPCKQTQTFTTYSD NQPAVTIQVFEGERAMTKDNNLLGTFDLTGIPPAPRGVPKIEVTF DJDANGILN	100
Motif 4	EIDALFEGIDFYTKISRARFEELCSDLFRSTLQPVEKALADAKMD KGSIHDIVLVGGSTRIPKIQNLLQNFFNGKTLNLSINPDEAVAYGA AVQAAILSG	100
Motif 5	EVKATAGDTHLGGEDFDNRLVNHFAEEFKRKYKKDLRTNPRAIR RLRTAAERAKRTLSSST	61
Motif 6	IGIDLGTTYSCVGVWQQGKVEIIANDQGNRTTPSYVAFT	39
Motif 7	KQDEHGYISRHFVRKYVLPKGHDINQVVSSLSSDGVLTITAPKT DKKAIEQRSI	54
Motif 8	KQDEHGYISRHFVRKYVLPKGHDINQVVSSLSSDGVLTITAPKT DKKAIEQRSI	68
Motif 9	VKGDKBVLQFBLGGGTFDVSEJTVKEGG	28
Motif 10	KLSEEDKERVRSECDECJKWLDSNQLAEKEEYEDKEKELESVCS PIMSKLHGGGG	55
Motif 11	DDPYDFPRPSRILDQQFGLGLDPEDLLAPVTPP	33
Motif 12	PVGYYRPWLSAAAQQDAGST	20
Motif 13	MGGGMPGGSCGQQAGGFGGARGGSGPTI	28
Motif 14	NIKLYVRRVFIKDECDDJJPEYLNFIKGVVDSEDLPLNISRELLQQ NKLIKVIRKKLVKKVL	62
Motif 15	FAFQAEVRQLMDLIINSLYSNKEIFLRELISNASDALDKIRYLSLT	46
Motif 16	MIGQFGVGFYSAFLVADKVVVVSKHNSDEQ	30
Motif 17	PIWTRDPDDISPQEYGEFYKALTGDWDDPLAVLHFNVEGQLSFR ALLFVPEGKP	54
Motif 18	LEIRIKPDKQEGTLTITDTGIGMTKQDLVNNLGTIAKSGTKAFL	44
Motif 19	RGTKIILHLKPDAKEFVEEDTIKEIVKKYSQFINYPIYLW	40
Motif 20	PIQQTGKPSKPVENKKEEKK	20
Motif 21	MFHSANGEEACSLKEYVKRMKPKQERIYFIAGESKEZVAKSPFV ERLKKKGFEVLYLVEPIDEY	64

Supplementary Table S3: Information of conserved motifs of DnaJ sequences in *Rhyzopertha dominica* searched by MEME Suite.

Motif ID	Sequence	Length (aa)
Motif 1	ILGVSRDASDEEIKKAYRKLALKYHPDKN	29
Motif 2	PEAEEKFKEISEAYEVLSDPKKRKIYDQY	29
Motif 3	LEIHIKPGMKAGTKITFPGEGDQGPDKEPGDIIFIJREKPHPVFKREGND	50
Motif 4	IIKPGTVKRIKGEGMPQYKNPSEKGBLIVNFDIEFPEQ	38
Motif 5	WLVDFYAPWCPPCQQLAPEWR	21
Motif 6	IVHTLKVSLEEALCGCTIKLK	21
Motif 7	RCSTCKGKG	9
Motif 8	DPEDLFREFFGGAGF	15
Motif 9	WTADDQQLLEEALKKFPGGTPERWDRIAECVGDRTKEDCM	40
Motif 10	VRIGKVDCEEDASLCEQQGVQSYPTIRLY	29
Motif 11	CCCCLCC	7

Supplementary Table S4: Details of bioassay data from the extreme high temperature treatment.

Treatment temperature (degree)	0 hour after treatment			24 hours after treatment		
	Average number of test insects	Average number of dead insects	Corrected mortality	Average number of test insects	Average number of dead insects	Corrected mortality
34	30	0	0	30	0	0
38	30	0	0	30	0	0
42	30	0	0	30	0	0
44	30	0	0	30	0	0
45	30	1.333	0.044	30	1.333	0.044
46	30	30	1	30	2	0.067
47	30	30	1	30	5.333	0.178
48	30	30	1	30	26	0.867
49	30	30	1	30	30	1
30 (control)	30	0	0	30	0	0

Supplementary Table S5: Details of bioassay data from the controlled nitrogen atmosphere treatment.

Treatment time (hours)	Average number of test insects	Average cumulative number of dead insects		Average cumulative mortality		Corrected mortality
		control	treatment	control	treatment	
24	30	0	0	0	0	0
48	30	0.333	4.333	0	0.144	0.144
72	30	0.333	13.333	0.011	0.444	0.438
96	30	0.333	20	0.011	0.667	0.663
120	30	0.333	25.333	0.011	0.844	0.842
144	30	0.333	27.667	0.011	0.922	0.921
168	30	0.333	29.667	0.011	0.988	0.988
192	30	0.667	30	0.022	1	1

Supplementary Table S6: Details of bioassay data from the phosphine fumigation treatment.

Concentration (g/m^3)	Log of concentration	Average number of test insects	Average number of dead insects	Corrected mortality	Probit value of mortality
0.15	-0.824	30	4.333	0.144	3.939
0.45	-0.346	30	15.333	0.511	5.028
0.75	-0.125	30	17.667	0.589	5.225
1.05	0.021	30	19	0.633	5.341
1.35	0.13	30	23	0.767	5.728
0 (control)	-	30	0	0	-

Supplementary Table S7: Details of bioassay data from the K-Obiol grain protectant treatment.

Concentration ($\mu g/cm^2$)	Log of concentration	Average number of total insects	Average number of dead insects	Corrected mortality	Probit value of mortality
0.0625	-1.204	30	1.667	0.056	3.407
0.125	-0.903	30	5	0.167	4.033
0.25	-0.602	30	10	0.333	4.569
0.5	-0.301	30	22.333	0.744	5.657
1	0	30	26	0.867	6.111
0 (control)	-	30	0	0	-
